# Impact of para aortic lymph node removal on survival following resection for pancreatic adenocarcinoma

**DOI:** 10.1186/s12893-023-02123-2

**Published:** 2023-08-01

**Authors:** Martin Sillesen, Carsten Palnæs Hansen, Stefan Kobbelgaard Burgdorf, Emilie Even Dencker, Paul Suno Krohn, Sophie Louise Gisela Kollbeck, Mogens Tornby Stender, Jan Henrik Storkholm

**Affiliations:** 1 Department of Organ Surgery and Transplantation, Copenhagen University Hospital, Rigshospitalet Blegdamsvej 9, Copenhagen, 2100 Denmark; 2 Center for Surgical Translation and Artificial Intelligence Research (CSTAR), Copenhagen University Hospital, Rigshospitalet, Denmark; 3 Institute of Clinical Medicine, University of Copenhagen, København, Denmark; 4 Department of Surgery, Aalborg University Hospital, Aalborg, Denmark; 5 Dep. of Surgery, Imperial College NHS trust, Hammersmith Hospital, London, UK

**Keywords:** Pancreatic cancer, Lymph station 16, Long term survival

## Abstract

**Introduction:**

For PDAC patients undergoing resection, it remains unclear whether metastases to the paraaortic lymph nodes (PALN+) have any prognostic significance and whether metastases should lead to the operation not being carried out. Our hypothesis is that PALN + status would be associated with short overall survival (OS) compared with PALN-, but longer OS compared with patients undergoing surgical exploration only (EXP).

**Methods:**

Patients with registered PALN removal from the nationwide Danish Pancreatic Cancer Database (DPCD) from May 1st 2011 to December 31st 2020 were assessed. A cohort of PDAC patients who only had explorative laparotomy due to non-resectable tumors were also included (EXP group). Survival analysis between groups were performed with cox-regression in a multivariate approach including relevant confounders.

**Results:**

A total of 1758 patients were assessed, including 424 (24.1%) patients who only underwent explorative surgery leaving 1334 (75.8%) patients for further assessment. Of these 158 patients (11.8%) had selective PALN removal, of whom 19 patients (12.0%) had PALN+. Survival analyses indicated that explorative surgery was associated with significantly shorter OS compared with resection and PALN + status (Hazard Ratio 2.36, p < 0.001). No difference between PALN + and PALN- status could be demonstrated in resected patients after controlling for confounders.

**Conclusion:**

PALN + status in patients undergoing resection offer improved survival compared with EXP. PALN + should not be seen as a contraindication for curative intended resection.

**Supplementary Information:**

The online version contains supplementary material available at 10.1186/s12893-023-02123-2.

## Introduction

During operation for pancreatic ductal adenocarcinoma (PDAC), adequate lymphadenectomy is important for correct staging. Based on the lymph node station definitions originating from the Japanese Research Society for Gastric Cancer (JRSG) [1], the International Study group on Pancreatic Surgery (ISGPS) currently recommends resection of stations 5, 6, 8a, 12b1, 12b2, 12c, 13a, 13b, 14a right lateral side, 14b right lateral side, 17a and 17b during pancreaticoduodenectomy (PD), and also stations 10, 11 and 18 during total pancreatectomy [2].

While the resection of these lymph node stations is generally accepted as common practice, considerable controversy remains about the resection of the paraaortic lymph nodes (PALN, Station 16b1). Data on lymphatic drainage pathways has shown that station 16b1 is an important node in the major lymphatic drainage route [3, 4], and studies have shown a reduced overall survival (OS) in patients with PALN metastasis (PALN+) [5, 6], and thus M1 classification according to the TNM system. It has been advocated that pancreatic resection should be aborted if frozen section during operation confirmed or preoperative radiographic workup suggested PALN +  [7]. This recommendation has, however, recently been challenged, and recent studies have concluded that while PALN + is an independent predictor of lower OS in line with what is observed in patients with local nodal involvement (N1 or N2 disease), PALN + patients still have a better OS than patients who only had explorative laparotomy or palliative surgery [8–10]. Furthermore, long-term survivors in the PALN + group can be identified [11].

There is currently no consensus on whether to proceed with resection in the PALN + setting, which is reflected in a lack of recommendation on this in the most recent ISPGS guidelines [2], where no strong recommendation could be given on resecting lymph node stations 8p, 14, 15 and 16b1 routinely. This was partly attributed to no survival benefit and increased number of complications during extended lymphadenectomy.

Using data from the Danish Pancreatic Cancer Database (DPCD), the purpose of this study was to retrospectively assess the OS of PDAC patients undergoing explorative laparotomy, pancreaticoduodenectomy (PD), or total pancreatectomies (TP) with selective harvesting and histological assessment of PALN. We hypothesized that PALN + would be associated with reduced overall survival compared to patients with resected tumors without PALN metastasis (PALN-), but still have a longer OS than patients who only had explorative laparotomy.

The rationale for the latter part of the hypotheses is that studies have identified long-term PALN + survivors with OS surpassing that of patients undergoing exploration only [8].

## Methods

The study includes all patients who were operated for PDAC between May 1st 2011 and December 31rd 2020 and subsequently included in the DPCD. Patients in whom PALN were removed for pathologic examination were divided into two groups, one with PALN+ (Metastasis to PALNs) and the other PALN- (no sign of metastasis). For comparison, patients with PDAC who only had explorative laparotomy or palliative operation (EXP) were included. The EXP group thus included patients planned for a curative operation, but with tumors found to be non-resectable perioperatively due to metastasis or carcinosis (M1 disease), locally advanced tumors, or tumors found to be non-resectable due to other causes (e.g., inflammation rendering tumors non-resectable). Of note, PALNs were not subjected to frozen section perioperatively, and PALN + status was not considered a contraindication to resection during the study period.

Patients, who underwent exploratory laparotomy with subsequent oncologic downstaging and successful surgical resection afterwards, were included in the relevant PALN groups according to the pathology report.

Patients, who had PD or TP without recorded harvesting of PALN, were excluded from further analyses.

In Denmark, pancreatic surgery is performed at four centers. The centers have comparable surgical approaches and overall outcomes. In general, the pylorus preserving approach is not practiced, with the PD performed as the Whipple’s procedure. Pancreaticojejunostomies are performed as described by Blumgart [12].

As for the extent of the PALN dissection, the standard procedures implied resection of the station 16b1 located in the supraaortic position caudal to the left renal vein but cranial to the origin of the Inferior Mesenteric Artery (IMA). In some procedures, parts of station16A2 from the overcrossing of the left renal vein over the aorta up to the origin of the Superior Mesenteric Artery (SMA) was also resected. This was, however, not independently reported in the pathology data.

Data were retrieved from the Danish Pancreatic Cancer Database (DPCD), which holds nationwide information on both surgically and non-surgically treated patients diagnosed with pancreatic, periampullary and duodenal adenocarcinoma. DPCD is linked with The National Pathology Register and the Register of Death.

The Study was approved by the DPCD board of governors as well as the Danish Capital Region Data Protection authority (Approval #P-2020-180). In compliance with Danish Law, informed consent and ethics board approval was not obtained due to the retrospective nature of this study. Specifically, the study adhered to national legislation as stipulated in the Data Protection Law (databeskyttelsesloven) of May 2018, amendment 1509 § 10, Sects. 1 and 2. Further information can be found on https://www.datatilsynet.dk/media/7952/videregivelsesvejledning.pdf.

We prepared the study in accordance with the “Strengthening the reporting of observational studies in epidemiology” (STROBE) guidelines [13].

### Statistical analyses

The study was planned as a survival analysis using overall survival (OS) as the primary endpoint. This was defined as time-to-event from the time of operation of either all-cause mortality or follow-up censoring which was set to November 1st, 2021.

For survival analyses, we used Kaplan-Meier estimates calculated for the EXP, PALN + and PALN- groups, respectively. Log-rank test was used for comparison of survival curves. A Bonferroni post-hoc test was used to correct for multiple comparisons.

To account for covariates, we used a cox-regression approach for both univariate and multivariate modelling with hazard ratios (HR). For the multivariate approach, two models were constructed. To compare OS between the EXP, PALN + and PALN- patients, we included covariates available in all three groups, including patient age, gender, Charlson Comorbidity Index (CCI) and pre or postoperative oncological treatment. To assess OS of patients after resection of tumor, we constructed an additional model corrected for shared covariates between the PALN + and PALN- groups. These included the above-mentioned covariates supplemented by tumor T and N stages as well as resection margin outcome (R0, R1 or R2). To assess the potential impact of the total number of harvested lymph nodes vs. the number of positive nodes harvested (lymph node ratio, LNR), a subgroup analyses was performed for resected patients where information on LNRs were available. As this ratio has been inconsistently registered in pathology records during the study timeframe, it was chosen to include this data point in a separate analyses.

Danish pancreas pathology definitions require a tumor free resection margin > 1 mm, which is in contrast to the international definitions of ≥1mm [14].

Data are presented as medians with interquartile range [IQR] or percentages, where appropriate. LNRs are presented as means. Kaplan-Meier survival estimates are presented with 95% confidence intervals.

Demographic and treatment related data in the PALN- and PALN + groups were compared using Mann-Whitney U-test or Chi-square test where appropriate.

We used the R statistical suite for the analyses [15]. A p-value of < 0.05 was considered statistically significant.

### Missing data

Missing data were considered missing at random (MAR). If applicable, the percentage of missing data points are shown alongside relevant values in the results tables. To assess the impact of missing data, a sensitivity analysis was performed comparing the original vs. imputed dataset in terms of cox regression results. For this purpose, the R package “MICE” was used for predictive mean matching. Supplementary Tables 1 and 2 hold information on the multivariate cox regression results.

## Results

In total, 1758 patients underwent operation for PDAC including 424 (24.1%) patients, who had explorative laparotomy. Of 1334 (75.8%) patients who had resection of their tumor, 158 (11.8%) patients had PALN removed for histologic evaluation, with 80% of these procedures carried out by a single center out of the 4 included pancreatic surgery centers (Copenhagen University Hospital, Rigshospitalet). In this group, 139 patients (88.0%) had PALN- and 19 patients (12.0%) had PALN+.

**Fig. 1:**
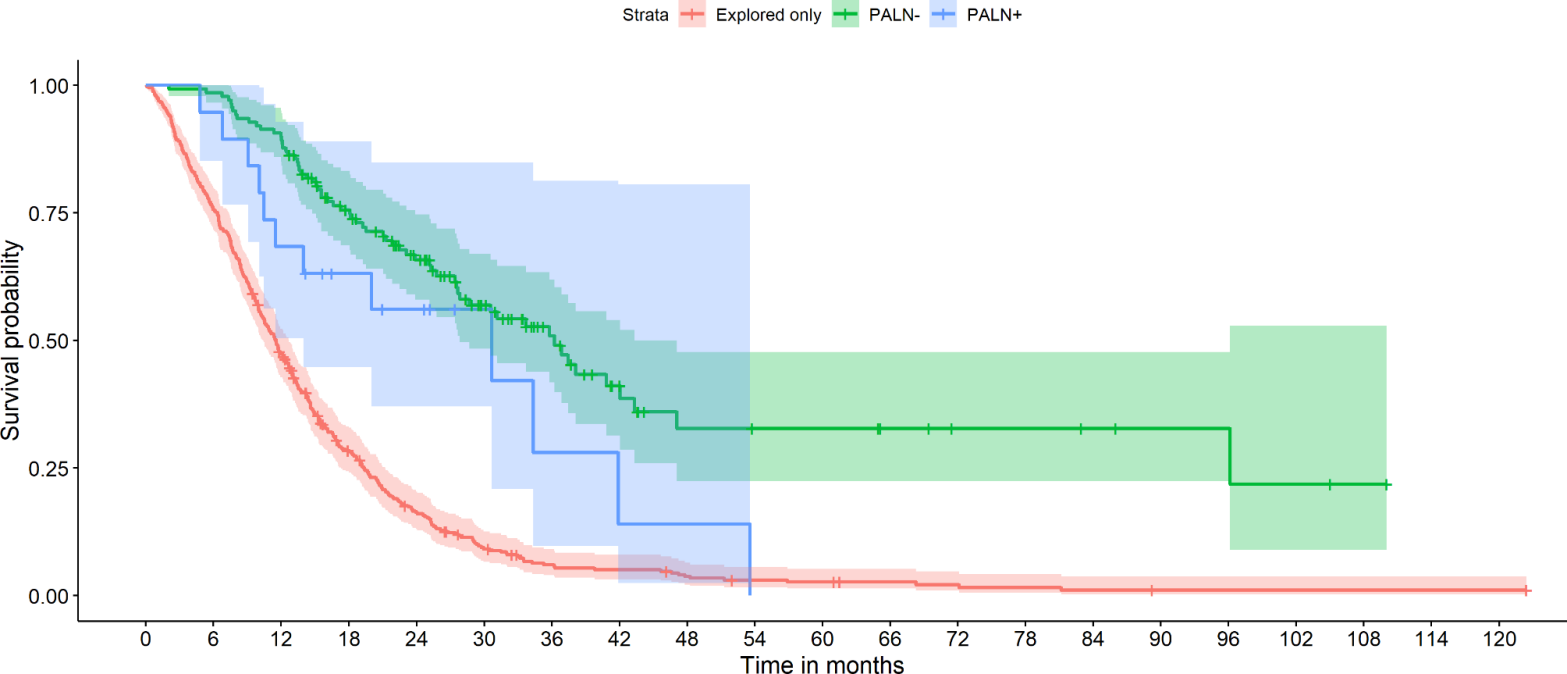
Overall survival of patients with metastases (PALN+) and no metastases (PALN-) to paraaortic lymph nodes, and patients who only had explorative laparotomy without lymph node resection (EXP). Log rank testing indicated a significant difference between PALN + and EXP survival curves (p = 0.04), but not between PALN + and PALN- survival curves (p = 0.10)

Table 1 provides an overview of demographic and treatment related variables between groups. Kaplan-Meier survival estimates is shown in Table 2 and graphically depicted in Fig. 1.

**Table 1:** Overview of demographic and treatment related variables between groups

		Paraaortic lymph node metastasis positive (PALN+) (N = 19)	Paraaortic lymph node metastasis negative (PALN-) (N = 139)	Explored only (EXP) (N = 424)	p-value*
Sex	Male (n,%)	11 (57.9%)	64 (46.0%)	239 (56.4%)	0.99
	Female (n,%)	8 (42.1%)	75 (54.0%)	185 (43.6%)	
Age	Median [Q1,Q3]	67.0 [57.2,73.6]	70.9 [61.2,76.3]	70.1 [63.4,76.0]	0.28
Charlson Comorbidity Index	Median [Q1,Q3]	1.00 [0,2.50]	1.00 [0,2.00]	1.00 [0,2.00]	0.47
Preoperative Chemotherapy	Yes (n,%)	2 (10.5%)	27 (19.4%)	66 (15.6%)	0.99
Postoperative Chemotherapy	Yes (n,%)	15 (78.9%)	113 (81.3%)	290 (68.4%)	0.99
Type of resection	Total Pancreatectomy (n,%)	6 (31.6%)	31 (22.3%)	1 (0.2%)	0.99
	Pancreaticoduodenectomy (n,%)	11 (57.9%)	95 (68.3%)	3 (0.7%)	
	Missing (n,%)	2 (10.5%)	13 (9.4%)	420 (99.1%)	
Pathology T stage	T1(n,%)	1 (5.3%)	13 (9.4%)	0 (0%)	0.99
	T2 (n,%)	8 (42.1%)	46 (33.1%)	1 (0.2%)	
	T3 (n,%)	8 (42.1%)	53 (38.1%)	1 (0.2%)	
	T4 (n,%)	0 (0%)	2 (1.4%)	0 (0%)	
	Missing (n,%)	2 (10.5%)	25 (18.0%)	422 (99.5%)	
Pathology N stage	N0 (n,%)	0 (0%)	33 (23.7%)	1 (0.2%)	0.99
	N1 (n,%)	4 (21.1%)	52 (37.4%)	1 (0.2%)	
	N2 (n,%)	13 (68.4%)	31 (22.3%)	0 (0%)	
	Missing (n,%)	2 (10.5%)	23 (16.5%)	422 (99.5%)	
Resection Margin	R0 (n,%)	15 (78.9%)	102 (73.4%)	1 (0.2%)	0.99
	R1 (n,%)	2 (10.5%)	8 (5.8%)	0 (0%)	
	R2 (n,%)	0 (0%)	3 (2.2%)	0 (0%)	
	Missing (n,%)	2 (10.5%)	26 (18.7%)	423 (99.8%)	
Survival status on end of follow-up	Alive	7 (36.8%)	74 (53.2%)	30 (7.1%)	
	Dead	12 (63.2%)	65 (46.8%)	394 (92.9%)	
Follow-up Time (months)	Median [Q1,Q3]	16.5 [11.0,26.3]	23.5 [15.1,33.6]	11.4 [6.35,18.3]	

*Comparison between PALN- and PALN + groups.

**Table 2:** Kaplan-Meier survival estimates

Group	Time	Survival estimate	95% confidence interval
EXP, (n = 424)	1 year	47.3%	42.8-52.3%
	2 years	16.3%	13.1-20.4%
	5 years	2.7%	1.4-5.2%
PALN- (n = 139)	1 year	89.9%	85.1-95.1%
	2 years	65.8%	58.0-74.7%
	5 years	32.7%	22.4-47.8%
PALN+ (n = 19)	1 year	68.4%	50.4-92.9%
	2 years	56.1%	37.1-84.9%
	5 years	NA	NA

EXP: Surgically explored only

PALN: Resected with paraaortic lymph node metastasis (PALN+) or without metastasis (PALN-).

NA: Data not available

Two-year OS was shorter in PALN + vs. PALN- (56.1% vs. 65.8%), but longer in PALN + vs. EXP (56.1% vs. 16.3%). Log rank testing indicated a significant difference between survival curves of PALN + vs. EXP (p = 0.04) but not between PALN + and PALN- (p = 0.10).

Results from the univariate and multivariate cox-regression models comparing OS in the EXP, PALN + and PALN- groups are shown in Table 3 and comparisons of resected patients (PALN + vs. PALN-) are shown in Table 4. Overall, PALN- status was associated with longer OS compared with PALN + in univariate modelling approaches, although this association could not be verified in the multivariate approach. As specified in the methods section, this approach used covariates available for all three groups.

**Table 3:** Univariate and multivariate cox regression comparing patients with paraaortic lymph node metastasis (PALN+), no paraaortic lymph node metastasis (PALN-) and patients undergoing surgical exploration only (EXP).

	Variable	Subtype	Hazard Ratio (95% conf. interval)	p-value
Univariate	Resection type*	PALN-	0.57(0.31–1.05)	**0.05**
		EXP	2.31(1.30–4.11)	**< 0.001**
	Demographic	Female sex	0.80(0.67–0.97)	**0.02**
		Age	1.01(1.00-1.03)	**< 0.001**
		Charlson Comorbidity Index	1.06(1.01–1.10)	**< 0.001**
	Oncology treatment	Preoperative chemotherapy	0.54(0.43–0.70)	**< 0.001**
		Postoperative Chemotherapy	0.42(0.34–0.51)	**< 0.001**
Multivariate	Resection type*	PALN-	0.50(0.27–0.93)	**0.03**
		EXP	2.36(1.32–4.20)	**< 0.001**
	Demographic	Female sex	0.85(0.71–1.03)	0.10
		Age	1.01(0.99–1.01)	0.39
		Charlson Comorbidity Index	1.03(0.99–1.07)	0.17
	Oncology treatment	Preoperative chemotherapy	0.43(0.33–0.55)	**< 0.001**
		Postoperative Chemotherapy	0.39(0.32–0.49)	**< 0.001**

*PALN- and EXP groups are compared with PALN + patients.

Multivariate models were corrected for the shown covariates

**Table 4:** Univariate and multivariate cox regression comparing resected patients with paraaortic lymph node metastasis (PALN+), or no paraaortic lymph node metastasis (PALN-).

	Variable	Subtype	Hazard Ratio (95% conf. interval)	p-value
Univariate	Resection type*	PALN-	0.55(0.29–1.01)	**0.05**
	Demographic	Female sex	0.72(0.46–1.14)	0.16
		Age	1.01(0.98–1.03)	0.51
		Charlson Comorbidity Index	1.06(0.97–1.16)	0.21
	Oncology treatment	Preoperative chemotherapy	0.70(0.38–1.30)	0.26
		Postoperative Chemotherapy	0.56(0.33–0.93)	**0.02**
	Tumor T-stage#	T2	1.42(0.54–3.73)	0.48
		T3	1.42(0.55–3.64)	0.47
		T4	1.01(0.12–9.19)	0.95
	Tumor N stage#	N1	1.46(0.76–2.80)	0.26
		N2	2.02(0.99–4.100)	**0.06**
	Resection Result§	R1	0.98(0.39–2.46)	0.97
		R2	1.95(0.47–8.08)	0.36
Multivariate	Resection type*	PALN-	0.77(0.36–1.63)	0.49
	Demographic	Female sex	0.87(0.48–1.56)	0.63
		Age	1.01(0.98–1.04)	0.59
		Charlson Comorbidity Index	1.10(0.98–1.25)	0.12
	Oncology treatment	Preoperative chemotherapy	0.85(0.33–2.23)	0.74
		Postoperative Chemotherapy	0.46(0.24–0.91)	**0.03**
	Tumor T-stage#	T2	0.90(0.40–3.10)	0.84
		T3	1.01(0.35–2.8)	0.98
		T4	2.06(0.04–5.9)	0.57
	Tumor N stage#	N1	2.39(1.05–5.44)	**0.04**
		N2	2.60(1.05–6.42)	**0.04**
	Resection Result§	R1	0.82(0.28–2.43)	0.72
		R2	2.86(0.61–13.48)	0.18

*Compared with PALN + group.

#Compared with T1 and N0, respectively

§Compared with R0 resection

Multivariate models were corrected for the shown covariates

Specifically, when all groups (PALN- and EXP) were compared with PALN + status, PALN- was associated with longer OS (HR 0.50, p = 0.03) in multivariate modelling as well as univariate models (HR 0.57, p = 0.05), whereas exploratory surgery only (EXP) was associated with reduced OS in both univariate (HR 2.31, p < 0.001) and multivariate (HR 2.36, p < 0.001) models.

This thus indicates a survival benefit associated with PALN- status when compared to both PALN + and EXP.

When PALN + was compared with PALN- only, including covariates available only for resected patients (Tumor T and N status as well as resection margin as described under methods), PALN- was associated with longer OS in univariate (HR 0.55, p = 0.05) although this association could not be confirmed in multivariate modelling (HR0.77, p = 0.49).

When the analysis was performed on the imputed dataset (Supplementary Tables 1 and 2), comparable results were obtained.

For comparative purposes, supplementary Fig. 1 depicts a Kaplan-Meier comparison of PALN + versus patients with PALN- status but N2 disease.

### Lymph node ratio subgroup analysis

A total of 82 (7.2%) resected patients had information available on LNR. Mean LNR was 0.25, with a mean total number of lymph nodes harvested of 22. In the multivariate cox regression model, LNR was found to be an independent predictor of OS (HR 1.21, p < 0.001). PALN- status remained non-significant when LNR was included as a covariate (HR 0.79, p = 0.51).

Information on LNR specifically for resected PALNs, was not available.

## Discussion

To our knowledge this is the largest study so far of OS in patients who systematically had extended extirpation of PALN during operation for PDAC. When assessed by a univariate approach, we found that PALN + status is an independent predictor of a shorter OS compared with PALN- status in patients following tumor resection. However, in the multivariate approach, PALN + status was not associated with a shorter survival. In this model, only histological N-status and whether the patient had adjuvant chemotherapy were independent predictors. As PALN + status is captured in the N-status, with 79% of PALN + patients having N2 disease, the value of the N-classification may thus be superior to PALN + status when assessing overall survival. These findings are in line with other studies [16].

In contrast, when PALN + status was compared with OS for EXP patients, PALN + status with curatively intended resection was associated with a significantly better OS than for EXP patients, which indicated that even with PALN + status, tumor resection still offers improved survival compared to no resection.

These results indicate that patients with PALN + status have survival in line with advanced T and N stages, especially N2 disease, even though PALN + is classified as M1 disease.

Our results seem to support findings in previous studies indicating that although PALN + status is a predictor of a shorter OS, this should not lead to deviation from planned tumor resection [6, 8] although this is still recommended in a recent study, [17].

Several differences between study methodologies may in part explain these findings. First, multiple studies have demonstrated a survival advantage of adjuvant chemotherapy following surgical treatment of PDAC [18–20], and this may have an impact on results in multivariate models. In our study, we have factored this into the multivariable models as opposed to a recent report concluding that PALN + status should result in aborted surgery [17]. Furthermore, although we have not been able to correct for this in the statistics, the choice of chemotherapeutic agents as well as treatment completion is likely also important [19].

Second, histological N-status appears to be a strong prognostic indicator [18, 19]. It should be emphasized that while the 8th edition of the American Joint Commission on Cancer (AJCC) guidelines on PDAC staging defines N1 status as 1–3 positive lymph nodes and N2 status as > 3 positive lymph nodes [21], this is critically dependent on the number of harvested lymph nodes during surgery. Studies have indicated a prognostic value of lymph node ratio (positive lymph nodes / total harvested lymph nodes) [22, 23] and recommend a minimum of 12 nodes harvested [24]. Although the results from the LNR subgroup analyses support these findings, it is important to underline that this was a post hoc analysis on a small subset of patients, and results should be interpreted with care.

Third, other factors such as vascular invasion [25] also have an independent prognostic relevance and could influence the results of the multivariate analyses, although a recent study by our group with data from patients in this study indicated that venous resection per se was not associated with OS [25].

Finally, it should also be noted that aside from a single meta-analysis [11], most studies are single center reports where the local patient population as well as surgical practices, techniques, and operative volumes may influence the outcomes.

The present study uses nationwide data on one of the largest PALN populations published to date. While our study adds to the knowledge concerning the prognostic value of PALN + status in PDAC, we cannot conclude on the potential value of implementing PALN removal as standard procedure during surgery.

When considering standardized resection of PALNs, potential complications should also be assessed. Studies have indicated that extended nerve plexus dissection around the major visceral arteries is associated with severe postoperative diarrhea [26]. This was to some extend pharmacologically controllable with acceptable long-term outcomes [27].

Our study has limitations that should be acknowledged. As is the case for any retrospective study, we can only observe associations and not conclude on causality. Furthermore, study findings are inherently dependent on the quality of the underlying data material, as well as the covariates chosen for the multivariate assessment. Although we have chosen a reasonable number of covariates, other relevant factors such as type of adjuvant chemotherapy, vascular resections and treatment completion rates would have been interesting to assess. We have, however, included T and N stages as the best studied factors associated with OS as a covariate.

Information on causes of mortality as well as postoperative complications related to retrieval of PALNs would have been of interest, including the important complication of chyle leak. These data points were, however, not available in the registries, and the study was thus fielded targeting the endpoint of OS only.

Although the underlying data derive from 4 dedicated centers of pancreatic surgery in a small national healthcare system with comparable outcomes and limited variations in the employed surgical techniques, minor technical variations between centers over time could potentially have an impact on results. This is a caveat when assessing factors such as non-resectability rates, which have improved over the study period.

Furthermore, while we have included information on LNRs, this data point was only available for a limited number of patients and not included in the main analyses. While the LNR data support previous reports indicating that LNR have prognostic value, the limited data introduces a risk of bias and the presented analyses on the potential impact of LNR should thus be analyzed with caution.

As the DPCD accrues data from a variety of Danish nationwide registries where patients can be cross-referenced using the unique Central Persons Registry (CPR) number, this traditionally offers a position of strength which is evidenced by the relatively low magnitude of the missing data in this study aside from LNR. It should furthermore be noted that the 158 patients of which 19 had PALN + status, is still a limited dataset. As such, statistical power issues may have an impact on the observed results.

During the last decade, more studies have indicated that some patients with PALN metastases may have better survival when compared to patients with other M-disease [8, 28] It may be time to consider this when reviewing the new AJCC classification. However, the existing ISGPS guidelines do not recommend standard removal of station 16 nodes. The discussion of centralization and the benefits of high volume centers [29], should in the future focus on recommendations regarding extended lymphadenectomies in a high volume setting.

In conclusion, we have demonstrated that PALN + patients have longer OS than EXP patients but failed to identify an OS difference between PALN + and PALN- in patients with resected tumors. With these results, it is important to underline that these data cannot support nor refute the resection of PALNs as a standard practice alone. This would require future studies on PALN resection in a protocolized setup with a relevant control group. While this should ideally be approached through a randomized setup, it is unlikely that such a study will be fielded due to ethical concerns.

## Electronic supplementary material

Below is the link to the electronic supplementary material.

Supplementary Material 1

Supplementary Material 2

## Data Availability

Data are available upon reasonable request from the Danish Regions Quality Control Program (Regionernes Kliniske Kvalitetsskringsprogram, RKKP, (www.rkkp.dk)
