# Supplementary material for: Impact of para aortic lymph node removal on survival following resection for pancreatic adenocarcinoma
Source: BMC Surg. 2023 Aug 1;23:214. doi: 10.1186/s12893-023-02123-2 (PMC10394933; doi:10.1186/s12893-023-02123-2)
Supplement: Supplementary file 1 — Supplementary Material 1 [file 12893_2023_2123_MOESM1_ESM.pdf]

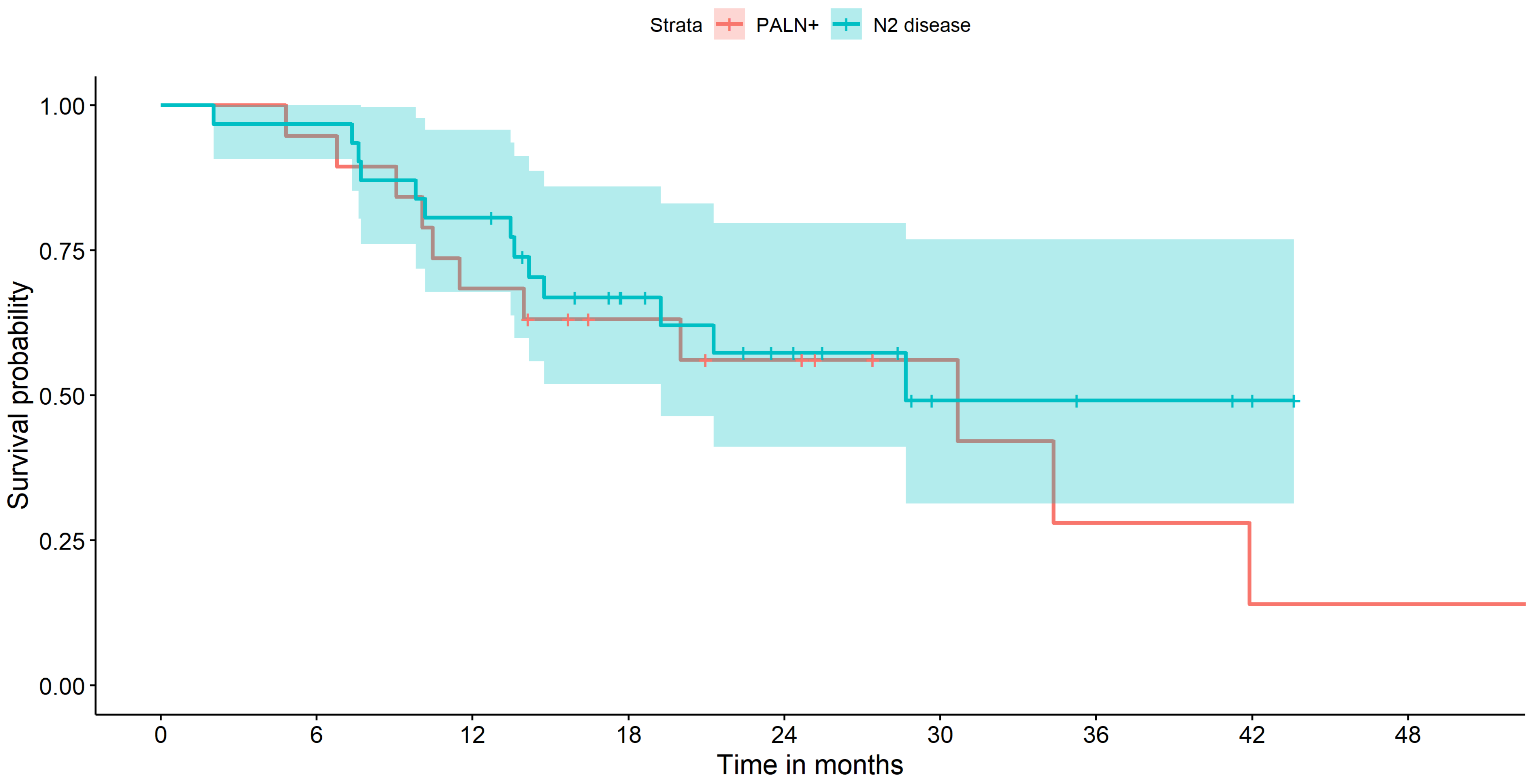

Supplementary figure 1: Kaplan-Meier survival curves comparing patients with paraaortic lymph node metastasis (PALN+) with patients without PALN metastasis but histological N2 disease.
As seen, PALN+ status survival was comparable to survival for N2 patients, which was confirmed by log-rank test (p=0.50)
