# Supplementary material for: Impact of para aortic lymph node removal on survival following resection for pancreatic adenocarcinoma
Source: BMC Surg. 2023 Aug 1;23:214. doi: 10.1186/s12893-023-02123-2 (PMC10394933; doi:10.1186/s12893-023-02123-2)
Supplement: Supplementary file 2 — Supplementary Material 2 [file 12893_2023_2123_MOESM2_ESM.docx]

|  | **Variable** | **Subtype** | **Hazard Ratio (95% conf. interval)** | **p-value** |
| --- | --- | --- | --- | --- |
| **Multivariate** | Resection type* | PALN- | 0.50(0.27-0.94) | **0.03** |
|  |  | EXP | 2.36(1.32-4.20) | **3.60x10^-3^** |
|  | Demographic | Female sex | 0.86(0.71-1.03) | 0.10 |
|  |  | Age | 1.00(0.99-1.01) | 0.39 |
|  |  | Charlson Comorbidity Index | 1.03(0.99-1.07) | 0.17 |
|  | Oncology treatment | Preoperative chemotherapy | 0.43(0.33-0.55) | **9.98x10^-11^** |
|  |  | Postoperative Chemotherapy | 0.40(0.32-0.49) | **2.00x10^-16^** |

**Supplementary Table 1:** Multivariate cox regression comparing patients with paraaortic lymph node metastasis (PALN+), no paraaortic lymph node metastasis (PALN-) and patients undergoing surgical exploration only (EXP).

*PALN- and EXP groups are compared with PALN+ patients.

Multivariate models were corrected for the shown covariates.

|  | **Variable** | **Subtype** | **Hazard Ratio (95% conf. interval)** | **p-value** |
| --- | --- | --- | --- | --- |
| **Multivariate** | Resection type* | PALN- | 0.57(0.29-1.11) | 0.10 |
|  | Demographic | Female sex | 0.82(0.50-1.33) | 0.41 |
|  |  | Age | 1.01(0.98-1.04) | 0.54 |
|  |  | Charlson Comorbidity Index | 1.07(0.97-1.19) | 0.18 |
|  | Oncology treatment | Preoperative chemotherapy | 0.83(0.39-1.74) | 0.61 |
|  |  | Postoperative Chemotherapy | 0.42(0.23-0.76) | **4*10^-3^** |
|  | Tumor T-stage# | T2 | 1.16(0.48-2.83) | 0.73 |
|  |  | T3 | 1.07(0.46-2.53) | 0.86 |
|  |  | T4 | 0.50(0.05-5.07) | 0.55 |
|  | Tumor N stage# | N1 | 1.44(0.73-2.86) | 0.29 |
|  |  | N2 | 1.88(0.90-3.93) | 0.09 |
|  | Resection Result§ | R1 | 1.18(0.49-2.87) | 0.70 |
|  |  | R2 | 2.17(0.74-6.36) | 0.16 |

**Supplementary Table 2:** Multivariate cox regression comparing resected patients with paraaortic lymph node metastasis (PALN+), or no paraaortic lymph node metastasis

(PALN-).

*Compared with PALN+ group.
#Compared with T1 and N0, respectively
§Compared with R0 resection.

Multivariate models were corrected for the shown covariates.
